# O067: Poorly processed reusable dispensers for surface disinfection tissues are a possible source of infection

**DOI:** 10.1186/2047-2994-2-S1-O67

**Published:** 2013-06-20

**Authors:** G Kampf, H von Baum, C Ostermeyer

**Affiliations:** 1 Bode Science Center, Bode Chemie GmbH, Hamburg, Germany; 2 Institute for Hygiene and Environmental Medicine, Ernst-Moritz-Arndt University, Greifswald, Germany; 3 Institut für Med. Mikrobiologie und Hygiene, Universität Ulm, Ulm, Germany; 4 Microbiology, Bode Chemie GmbH, Hamburg, Germany

## Introduction

Reusable surface disinfectant (SD) tissue dispensers are used in hospitals in many countries because they allow immediate access to soaked tissues for targeted surface decontamination.

## Objectives

We determined the frequency of contaminated SD solutions in reusable dispensers and the ability of isolates to multiply in different formulations.

## Methods

Dispensers with different SD were randomly collected from healthcare facilities. Solutions were investigated for bacterial contamination using standard microbiological methods. Isolates of the same species were investigated by pulsed-field gel electrophoresis (PFGE) for clonal identity. The efficacy of two SD was determined in suspension tests (EN 13727) under dirty conditions against two isolated species directly from a contaminated solution or after 5 passages without selection pressure in triplicate. Fresh use solutions of four different types of SD were contaminated with a fresh dispenser isolate to determine its survival or multiplication over 28 days.

## Results

66 dispensers containing SD solutions with surface-active ingredients were collected from 15 healthcare facilities. 28 dispensers from nine healthcare facilities were contaminated with approximately 10^7^ cells per mL of *Achromobacter species 3* (9 hospitals), *Achromobacter xylosoxidans* or *Serratia marcescens* (1 hospital each). Clonal non-identity was shown for 8 of 9 *Achromobacter species 3* isolates. In none of the hospitals dispenser processing was adequately performed. Isolates regained susceptibility to the SD after five passages without selection pressure, for example against *Achromobacter species 3* with a mean log_10_-reduction of 0.06 initially and 2.37 after 5 passages (Incidin plus 0.5% for 60 min). Adapted and passaged cells were equally able to multiply in different formulations from different manufacturers with surface-active ingredients at room temperature within 7 days to a cell count of 10^7^ bacteria per mL, only a formulation with additional aldehyde was able to completely kill the contamination.

## Conclusion

Neglecting adequate processing of tissue dispensers has contributed to frequent and heavy contamination of use-solutions of SD based on surface active ingredients.

## Disclosure of interest

G. Kampf Employee of Bode Chemie GmbH, Hamburg, Germany, H. von Baum: None declared, C. Ostermeyer Employee of Bode Chemie GmbH, Hamburg, Germany.

